# Exploring the attitudes and practices of female doctors towards cervical cancer screening in primary health care centers

**DOI:** 10.25122/jml-2022-0344

**Published:** 2023-05

**Authors:** Ahmed Aldarmahi, Hanan Alzahrani, Sulafah Alqutub, Faisal Alzahrani

**Affiliations:** 1. College of Science and Health Professions, King Saud bin Abdulaziz University for Health Sciences, Jeddah, Saudi Arabia; 2. King Abdullah International Medical Research Center, Jeddah, Saudi Arabia; 3. Primary Health Care, Ministry of Health, Jeddah, Saudi Arabia; 4. Faculty of Medicine, University of Jeddah, Jeddah, Saudi Arabia; 5. Department of Clinical Laboratory Sciences, College of Applied Medical Sciences, Imam Abdulrahman bin Faisal University, Dammam, Saudi Arabia

**Keywords:** knowledge, attitude, KAP study, Pap smear test, primary healthcare, women's health

## Abstract

Cervical cancer is a significant cause of female mortality worldwide, and early detection through regular screening is crucial for reducing mortality rates. However, in developing countries, the uptake of Pap smear tests (PST) is low, mostly due to cultural and social factors and a lack of knowledge. This cross-sectional study assessed knowledge, attitude, and practice of cervical cancer screening among practitioners working at primary healthcare centers in Saudi Arabia. Furthermore, the study aimed to identify the potential barriers that prevent female physicians from performing cervical cancer screening tests. A self-administrated, well-structured questionnaire was used to survey 95 female physicians, including residents, specialists, and consultants in several primary health care centers (PHCCs) in Jeddah managed by the Ministry of Health during September 2019. The results showed that 80% of participants knew about cervical cancer, and 97.8% were aware that PST is a screening tool. However, only 47% advised female patients to get tested for cervical cancer. The factors identified as barriers to test uptake included asymptomatic females (34%), lack of time on the part of the practitioner (24%), and a lack of evidence for risk factors (23%). Additionally, only 42.2% of the participating physicians had undergone a Pap smear test themselves. The study highlights the need for further research to assess HPV status in the population and explore the correlation between circumcision and cervical cancer, as well as polygamy and cervical cancer. The findings suggest that while a good level of knowledge about cervical cancer exists, there is a need to improve compliance with cervical cancer screening guidelines among female physicians in Saudi Arabia.

## INTRODUCTION

According to a worldwide analysis, cervical cancer has become the second most common malignancy and the second leading cause of death in women of reproductive age [1]. In 2021, the annual incidence and mortality rates for women were 7.5 per 100,000 and 2.2 per 100,000, respectively, after adjusting for age. The American Cancer Society estimates that the US alone will have 13,800 (0.8%) new cases of cancer and 4,290 (0.7%) cancer-related deaths [2]. The countries with the lowest ranking on the Human Development Index [3] have the highest incidence and mortality rates of cervical cancer, accounting for nearly 604,127 new cases and 341,831 deaths [4]. Despite the high mortality, cervical cancer is preventable by implementing health strategies like vaccination, screening programs for women, and initiation of effective treatment for precancerous cervical lesions. It is a great public health concern as it affects females of a wide range of reproductive age groups. Around 95% of women with stage I and II, as well as 60% with stage III cervical cancers, can be cured with surgery and chemotherapy [5].

Several risk factors have been associated with cervical cancers, such as venereal transfer of infectious agents like human papillomavirus (HPV) [6,7], herpes simplex virus type 2 (HSV-2), Chlamydia trachomatis [8]. Other factors include sexual history, smoking [9], compromised immune system, long-term use of oral contraceptives, and others [10]. Furthermore, abnormal vaginal bleeding (inter-menstrual, post-coital, or post-menopausal) is a common symptom in about 60% of cases [11]. Other symptoms like dyspareunia and pelvic pain are uncommon and indicate a more advanced lesion. As a complication, cervical cancer can lead to obstructive uropathy, which may present with symptoms such as hematuria.

Reducing cancer incidence and mortality is the main purpose of advocating preventive measures, such as vaccination, avoiding skin-to-skin contact by using barriers to prevent HPV transmission, and, most importantly, screening for cervical cancer using PST. HPV vaccination provides primary prevention against cervical cancer and is available in three types: the first type covers HPV types 16/18, the second type covers types 11, 6, 16, and 18, and the third type covers types 31, 33, 45, 52, and 58 [6,7]. The standard process for diagnosing cervical cancer involves a Pap smear for cytology followed by a colposcopy-directed biopsy, and then conization if the lesion is not visible, there is a high-grade smear, or the colposcopy was unsatisfactory. The lesion is then staged and graded using the International Federation of Gynecology and Obstetrics (FIGO) staging system, the most commonly used method for clinical staging [5]. The grading of the lesion requires either a cytological sample or tissue for biopsy. Cervical cancer screening guidelines recommend either cytology alone or primary HPV testing and cytology as co-testing [12].

The age-standardized incidence rate of cervical cancer in Saudi Arabia is 2.8%, and the mortality rate associated with it is 1.6% [12]. Cervical cancer is considered the ninth most frequent cancer among Saudi women aged 15 to 65 years old and the sixth cause of mortality. HPV infections account for 89% of cervical cancers in Saudi Arabia, with types 16/18 accounting for 78.7% of cases [7]. Although there are several published studies regarding the level of knowledge, attitude, and practice toward cervical cancer awareness among women in the community, few studies have explored knowledge and adherence to PST among physicians. A study conducted in Bahrain and published in 2018 measured the level of awareness and the number of females who carried out cervical cancer screening at primary health care centers (PHCCs). The study found that nearly 64% heard about Pap smear, while only 3.7% heard about HPV [13]. Similarly, Almobarak *et al*. found that half of the Sudanese women who participated in their study never heard of PST despite having a university or higher education level [14]. Another study in Nigeria found that 40.5% of the respondents, including doctors and nurses, had better knowledge (51.2%) than non-clinical staff [15]. A study conducted in Riyadh in 2017 included 507 participants and showed that nearly 60% of the women heard of the screening test, but 75% had never undergone PST [16]. In addition, a study conducted on 200 doctors working in King Abdul-Aziz University Hospital (KAUH), Jeddah, revealed that while more than two-thirds of the gynecological specialists knew about cervical cancer screening, the same could not be said about participants from other non-gynecological specialties. The study also found that the number of female physicians who underwent screening themselves was only 37.1% and 23.8% from gynecological and non-gynecological specialties, respectively [17]. These findings further highlight the lack of knowledge and inability to perform cervical cancer screening among female physicians and women.

### Rationale

Women's health is an integral component of primary healthcare practice. Cervical cancer is a preventable malignancy that affects women worldwide. Despite a few studies being conducted to identify factors influencing the lack of knowledge and awareness regarding cervical cancer screening among physicians and the general population, there is a lack of recent data in the western region, with the last known survey being conducted in 2018, limited to a tertiary care setup [18]. Thus, our study aimed to evaluate the knowledge, attitude, and practice of cervical cancer screening among female physicians who provide care to women at PHCCs. The results of this study can help identify the current situation and factors influencing it and assist in designing effective interventional programs such as awareness campaigns, education, and policy changes. Additionally, the study assessed the level of compliance to cervical cancer screening among female physicians themselves and identified the barriers that hinder the implementation of Pap smear screening tests.

## MATERIAL AND METHODS

### Study design and population

This cross-sectional study was conducted at primary health care centers (PHCCs) in Jeddah, the second-largest city in the Kingdom of Saudi Arabia (KSA), in the Western region in the governorate of Makkah Al-Mokarramah. The health services delivered to the community are free of cost through PHCCs under the Ministry of Health (MoH). The study population included certified female doctors, including general physicians, specialists, and consultants, regardless of their marital status and nationality, who worked at PHCCs in Jeddah and consented to participate in the study.

### Study location of PHCCs

The PHCCs in Jeddah are distributed over a wide geographical area and divided into five major regions: the middle, northern, eastern, western, and southern regions. Each region has its respective governmental hospital under the MoH, and each hospital manages its PHCCs distributed all over its catchment area, which includes many districts. All these government hospitals were approached to collect the data and include:

- King Fahad General Hospital with PHCCs in Al-Bawadi, Al-Naiem, Al-Nahdha, Al-Rabwah, Al-Marwah, Al-Safa one, Al-Safa two, Al-Aziziah, Al-Hamrah, Al-Ruwais, Al-Fyisaliah and Meshrefah districts.
- King Abdulaziz Hospital with PHCCs in Al-Mahjar, Al-Thaleba, Golil, Al-Quriat, Al-Bald, and Madain Al-Fahad districts.
- Althager Hospital with PHCCs in Kilo-13, Kilo-14, Om Al-Salam, Prince Abdulmajeed, and Al-Montazahat districts.
- The East Jeddah Hospital with PHCCs in Al-Solimaniah, Sharg Al-Khat, Al-Twfeeq, Al-Jamah, Al-Rawabi, Al-Naseem, Al-Rehab, and Briman districts.
- King Abdullah complex in the northern region with PHCCs in Al-Murjan, Abhar, Al-Sheraa, Al-Ryan, Al-Majed, Khalid, Al-Riyadh, and Al-Wafaa districts.

### Sample size

The sample size for the study was calculated after obtaining the total number of female physicians working at PHCCs in Jeddah using the RaoSOFT website with a margin of error of 5%, confidence level (CI) of 95%, and response rate of 75%. Convenience sampling was used to select the study participants from the population of 150 female doctors employed at PHCCs in Jeddah, considering that certain physicians may be unwilling or otherwise occupied.

### Data collection techniques

The participants completed a self-administered questionnaire in English, which consisted of four main domains: sociodemographic details (age, job title, marital and smoking status); closed-ended questions to assess knowledge, a five-point Likert scale to assess attitudes, and questions regarding their practices with patients and personal screening. The questionnaire also assessed barriers to practicing Pap smear screening. After the questionnaire was designed, a pilot study was conducted to test the validity and reliability of the questionnaire among ten female doctors who were not included in the study sample. This pilot study was performed to estimate the time needed to complete the questionnaire, which was less than five minutes. The questionnaire was distributed to female doctors willing to participate in the study during working hours at PHCCs. Each participant filled out the questionnaire in a face-to-face survey. No soft copy of the questionnaire was distributed to avoid contamination in the study population. The dependent variables were knowledge, attitude, and practice. The independent variables were age, job titles, marital status, and smoking history.

The collected data were coded and entered into Statistical Package for Social Sciences (SPSS) version 24.0 for analysis. Descriptive statistics were used to summarize the data and calculate the mean values ± standard deviation (SD) for continuous variables. Various statistical analysis methods were utilized, including percentage distribution, descriptive statistics, and a Chi-square test for independence between two categorical variables. The reliability of the questionnaire was assessed using Cronbach's alpha, which was calculated at 0.87, indicating a high standard of response reliability. A p-value less than or equal to 0.05 was considered significant.

## RESULTS

**Figure 1.**
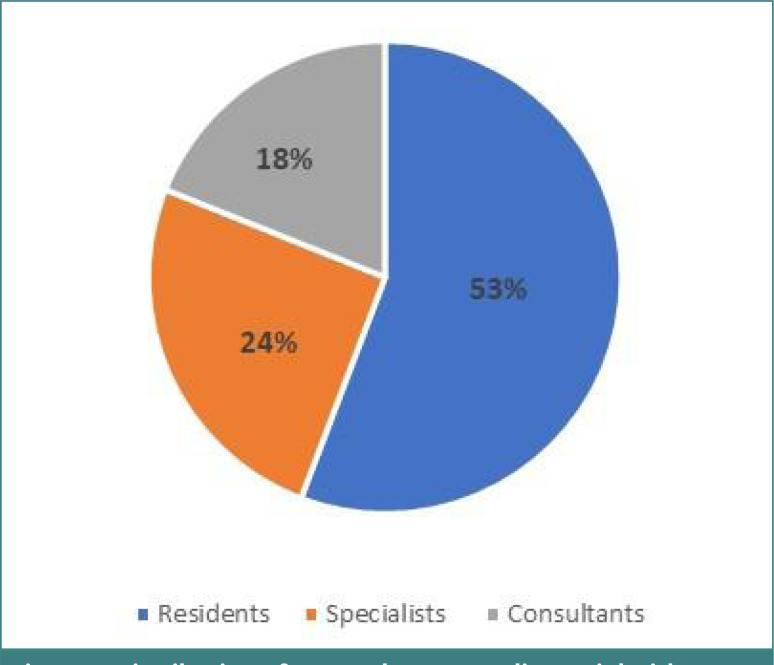
Distribution of respondents according to job titles

The questionnaire covered different dimensions, including demographic details and basic characteristics of the respondents’ professional backgrounds. The final dataset included responses from 95 female doctors with a mean age of 38.6±7.51 years, comprising residents, specialists, and consultants working at PHCCs. Figure 1 shows the distribution of respondents according to their job titles.

The sociodemographic details of the respondents were also considered with respect to their job titles were also considered. Most respondents were married (81%) and non-smokers (94.7%). Additional information on age, marital status, and smoking habits according to job titles can be found in Table 1.

**Table 1.** Sociodemographic characteristics of the respondents

Variable	Occupational title			Total N (%)
	Residents (53)	Specialists (24)	Consultants (18)	
	95 (100%)			
Mean age (years)	36.7	37.2	40	.512
**Marital status**				
Married	41 (77.4)	19 (79.2)	16 (88.8)	77 (81)
Divorced	4 (7.5)	0 (0)	1 (5.6)	5 (5.3)
Widowed	2 (3.7)	0 (0)	0 (0)	2 (2.1)
Single	6 (11.3)	5 (20.8)	1 (5.6)	12 (12.6)
**Smoking status**				
Smoker	4	1	0	5 (5.3)
Non-smoker	49	23	18	90 (94.7)
**Total**				95 (100)

Another section of the questionnaire assessed knowledge of cervical cancer (consisting of ten questions). Overall, physicians recognized the importance of PST as a screening tool (97.9%), followed by an awareness of risk factors (89.5%). The affirmative responses to this section suggested that 77.2% of the respondents had a good knowledge of cervical cancer ranging from information regarding mortality, curability, screening tests, their importance, risk factors, and more. However, 14% of participants reported being unaware of certain aspects of cervical cancers, and 8.6% declared a complete lack of awareness. Detailed responses for this section can be found in Table 2.

**Table 2.** Physicians’ responses to statements assessing knowledge of cervical cancer

	Statement	N (%) = 95 (100%)		
		Yes	No	I do not know
1	Cervical cancer is a leading cause of death among women	57 (60)	30 (31.6)	8 (8.4)
2	Cervical cancer is a curable disease	71 (74.7)	11(11.6)	13 (13.7)
3	The Pap smear is a screening tool used to detect precancerous lesions on the cervix	93 (97.9)	2 (2.1)	0 (0)
4	Screening for cervical cancer using a Pap smear typically starts three years after a person becomes sexually active or at 21 years of age	62 (65.3)	17 (17.9)	16 (16.8)
5	Cervical cancer in its early stage has no symptoms; vaginal bleeding	52 (54.7)	36 (37.9)	7 (7.4)
6	Almost all cases of cervical cancer are caused by infection with the human papillomavirus (HPV)	79 (83.2)	8 (8.4)	8 (8.4)
7	Being a sexually active female in the reproductive age range is a risk factor	74 (77.9)	16 (16.8)	5 (5.3)
8	Multiple sexual partners are a risk factor	85 (89.5)	5 (5.3)	5 (5.3)
9	Sexually transmitted disease is a risk factor	85 (89.5)	4 (4.2)	6 (6.3)
10	Low immunity status is a risk factor; HIV, immune deficiency disease, or receiving immunosuppressant agents	76 (80)	5 (5.3)	14 (14.7)

The second part of the questionnaire evaluated attitudes toward cervical cancer screening by presenting two statements and asking participants to respond using a Likert scale. Nearly 84% of doctors strongly agreed that screening for cervical is essential for women's health. Similarly, 81% of female physicians strongly agreed on beginning a screening program in the community. Detailed responses for this section can be found in Table 3.

**Table 3.** Attitude of participants regarding the implementation of cervical cancer screening

Statement	Strongly agree	Agree	Neutral	Disagree	Strongly disagree
Cervical cancer screening is essential for women's health	80 (84.2%)	11 (11.6%)	4 (4.2%)	0	0
A cervical screening program should be started in the community	77 (81.1%)	16 (16.8%)	2 (2.1%)	0	0

**Figure 2.**
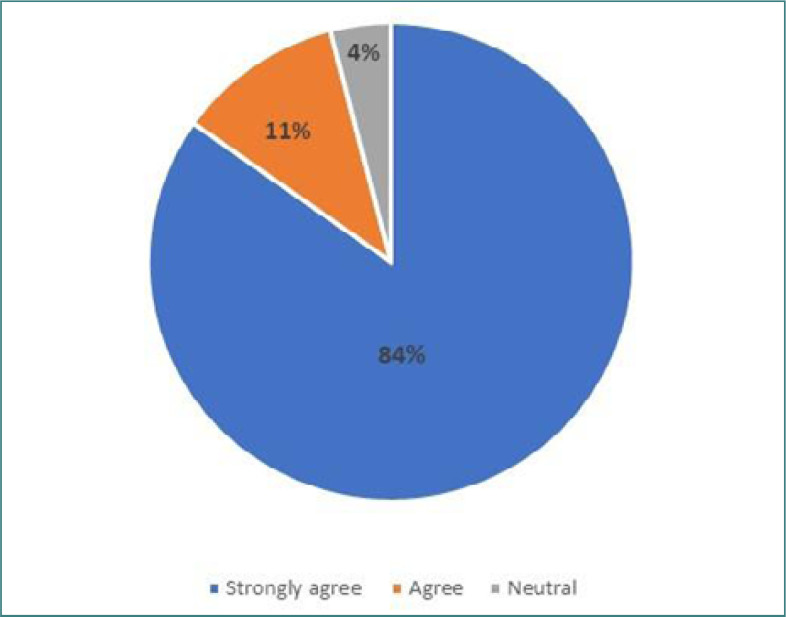
Participants' perception of the importance of cervical cancer screening for women's health

**Figure 3.**
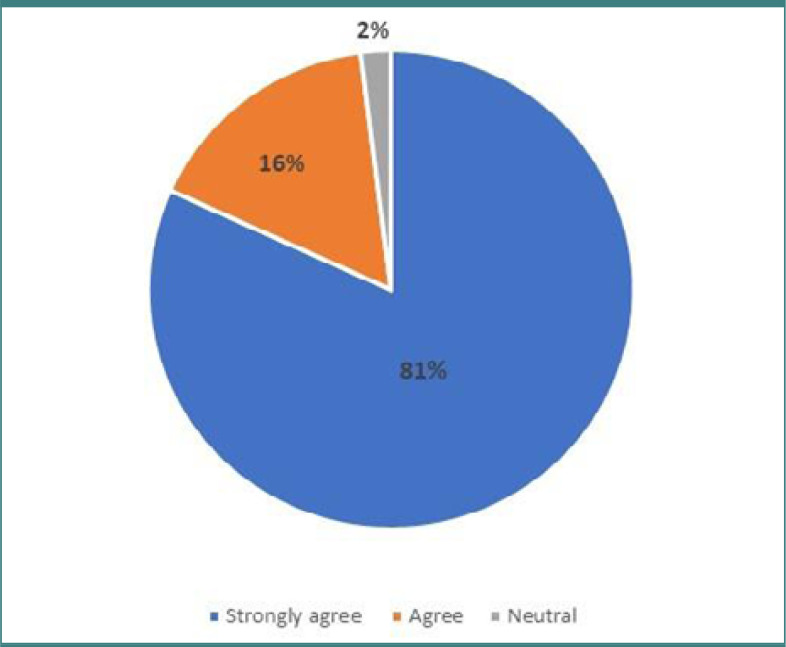
Participants’ attitudes toward starting a cervical screening program in the community

Figure 2 illustrates physicians' responses regarding the importance of cervical cancer screening, while Figure 3 displays the number of physicians who believe that screening for cervical cancer is necessary in the community.

The fourth section of the questionnaire consisted of two parts: one focused on the physicians' practice of referring patients for a PST, and the other on their own experiences of undergoing the procedure. The results showed that approximately 49.5% of the doctors referred their patients for cervical cancer screening, with only 12.6% adhering to the recommended three-year interval. In contrast, only 32% of the female physicians had undergone the screening themselves, with a mere 6% repeating it regularly (Table 4).

**Table 4.** Participants’ attitudes towards referring patients and undergoing cervical cancer screening

	Statement	Yes	No
1	I refer my patients to get a Pap smear as a screening test	47 (49.5%)	48 (50.5%)
2	I refer my patients to get a Pap smear every three years	12 (12.6%)	83 (87.4%)
3	I do not have time to refer the patient for a Pap smear	23 (24.2%)	27 (75.8%)
4	They do not have any symptoms or complaints	47 (49.5%)	48 (50.5%)
5	They are not at risk for cervical cancer	33 (34.7%)	62 (65.3%)
6	Have you ever received a Pap smear test?	31 (32.6%)	64 (67.4%)
7	I get a Pap smear test at regular intervals every three years	6 (6.3%)	89 (93.7%)
8	I do not have time	45 (47.4%)	50 (50.2%)
9	I am not at risk for cervical cancer	25 (26.3%)	70 (73.7%)
10	The procedure is painful	20 (21.1%)	75 (78.9%)
11	I do not like to know if I have cervical cancer	12 (12.6%)	83 (87.4%)
12	I do not have any gynecological symptoms	44 (46.3%)	51 (53.7%)

**Figure 4.**
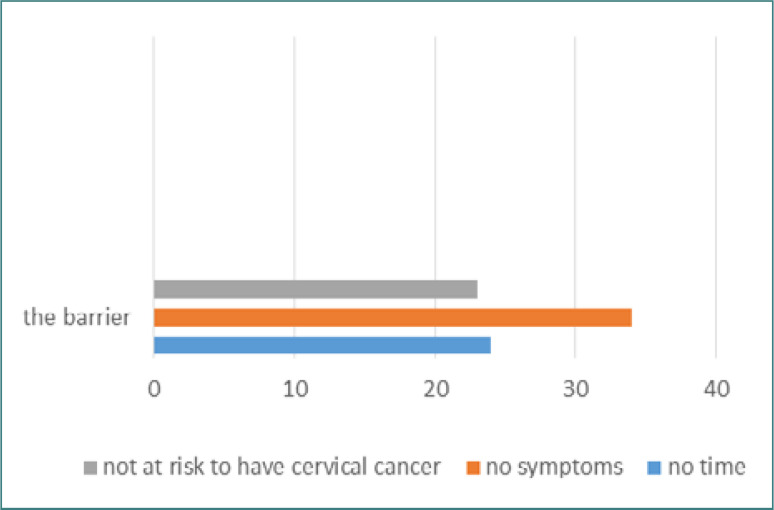
Barriers that prevent physicians from referring women for Pap tests

**Figure 5.**
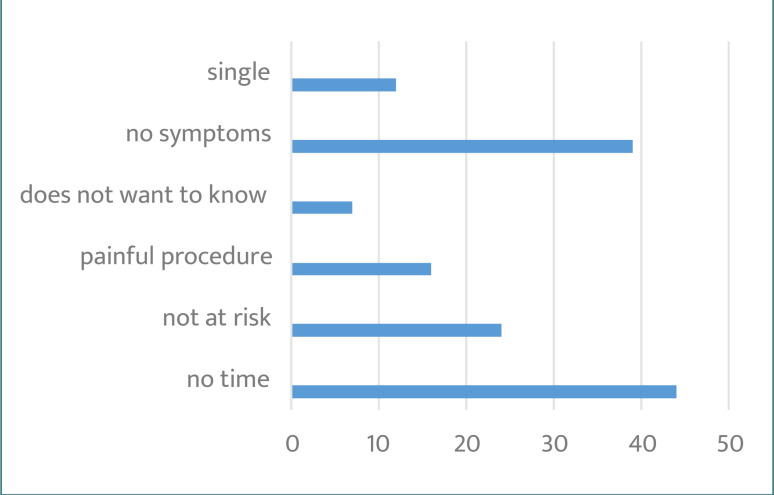
Barriers preventing physicians from getting a Pap smear test (PST)

The study also investigated factors that hindered referrals for PST and found that 47% of physicians believed women visiting PHCCs had no gynecological symptoms, while 24% reported a lack of time to perform the test. Additionally, 34% considered women visiting the clinic not at risk and not in need of a referral. Details on the barriers that prevent referring women for PST are presented in Figure 4. The questionnaire also asked about physicians' experiences with Pap smear testing and revealed that 47% of respondents had no time, 46% considered themselves asymptomatic, and 26% did not believe they were at risk. Figure 5 provides a list of factors that prevented physicians from receiving a Pap smear test.

A comparison of data collected from the three physician groups, categorized by job titles, revealed that specialist gynecologists generally had a higher level of knowledge, as indicated by the scores on the questionnaire. However, they showed lower scores when it comes to knowledge of causative factors and risk factors like low immunity levels, as assessed by statements six and ten (Table 5A).

**Table 5A.** Comparison of participants' knowledge about cervical cancer based on responses to survey questions

	Statement	Occupational title (correct answers)			P value
		Consultant	Specialist	Residents	
1	Cervical cancer is a leading cause of death in women	11 (61.1%)	15 (62.5%)	31 (58.5%)	0.98
2	Cervical cancer is a curable disease	13 (72.2%)	20 (83.3%)	38 (71.7%)	0.85
3	The Pap smear is a screening tool used to detect precancerous lesions on the cervix	18 (100%)	24 (100%)	51 (96.2%)	0.44
4	Screening for cervical cancer using a Pap smear typically starts three years after a person becomes sexually active or at 21 years of age	12 (66.7%)	22 (91.7%)	28 (52.8%)	0.020
5	Cervical cancer in its early stage has no symptoms; vaginal bleeding	11 (61.1%)	15 (62.5%)	26 (49.1%)	0.45
6	Almost all cases of cervical cancer are caused by infection with the human papillomavirus (HPV)	18 (100%)	22 (91.7%)	39 (73.6%)	0.044
7	Being a sexually active female in the reproductive age range is a risk factor	15 (83.3%)	20 (83.3%)	39 (73.6%)	0.69
8	Multiple sexual partners are a risk factor	18 (100%)	24 (100%)	43 (81.1%)	0.065
9	Sexually transmitted disease is a risk factor	17 (94.4%)	24 (100%)	44 (83%)	0.19
10	Low immunity status is a risk factor; HIV, immune deficiency disease, or receiving immunosuppressant agents	17 (94.4%)	19 (79.2%)	40 (75.5%)	0.44

The responses collected from consultants, specialists, and residents working at PHCCs indicate that a majority of consultants (94.4%) considered cervical cancer screening important for women's health, while most specialists advocated for a community-based program to increase screening uptake. However, consultants did not share this view with the same enthusiasm (77.8%). Table 5B provides further details of the comparison between the three groups.

**Table 5B.** Comparison of attitudes regarding screening for cervical cancer between the physician groups

Statement	Attitude	Occupational title (correct answers)			P value
		Consultant	Specialist	Residents	
Cervical cancer screening is essential for women's health	Strongly Agree	17 (94.4%)	22 (91.7%)	41 (77.4%)	0.52
	Agree	1 (5.6%)	2 (8.3%)	8 (15.1%)	
	Neutral	0 (0%)	0 (0%)	4 (7.5%)	
A cervical screening program should start in the community	Strongly Agree	14 (77.8%)	22 (91.7%)	41 (77.4%)	0.48
	Agree	3 (16.8%)	2 (8.3%)	11 (20.8%)	
	Neutral	1 (5.6%)	0 (0%)	1 (1.9%)	

The study included a set of questions to inquire about the doctors' practical approach when advising patients and themselves about cervical cancer screening. Questions 1 through 5 focused on patient referrals, while questions 6 through 13 asked about the factors that influence the doctors' approach to getting a screening test for themselves. The responses are presented in Table 5C, showing that consultant gynecologists refer their patients more frequently than the other two groups and follow their own advice by getting screened.

**Table 5C.** Comparison of participants' knowledge about cervical cancer based on responses to survey questions

	Statement	Occupational title (correct answers)			P value
		Consultant	Specialist	Residents	
1	I refer my patients for Pap smear screening tests starting at the age of 21 years	10 (55.6%)	13(54.2%)	24 (45.3%)	0.65
2	I refer my patients for a Pap smear every three years	2 (11.1%)	5 (20.8%)	5 (9.4%)	0.37
3	I do not have time to refer the patient for a Pap smear	2 (11.1%)	10(41.7%)	11 (20.8%)	0.049
4	They do not have any symptoms or complaints	5 (27.8%)	11 (45.8%)	31 (58.5%)	0.07
5	They are not at risk for cervical cancer	5 (27.8%)	6 (25%)	22 (41.5%)	0.29
6	Have you ever received a Pap smear test	12 (66.6%)	8 (33.3%)	11 (20.8%)	0.002
7	I have a Pap smear test at regular interval every three years	2 (11.1%)	1 (4.2%)	3 (5.7%)	0.63
8	I do not have time	9 (50%)	11 (45.8%)	25 (47.2%)	0.96
9	I am not at risk for cervical cancer	4 (22.2%)	6 (25%)	15 (28.3%)	0.87
10	The procedure is painful	4 (22.2%)	4 (16.7%)	12(22.65%)	0.83
11	I do not like to know if I have cervical cancer	2 (11.1%)	3 (12.5%)	7 (13.2%)	0.97
12	I do not have any gynecological symptoms	5 (27.8%)	14 (58.3%)	25 (47.2%)	0.14
13	I am single, never married	0 (0%)	5(20.8%)	6 (11.3%)	0.11

**Figure 6.**
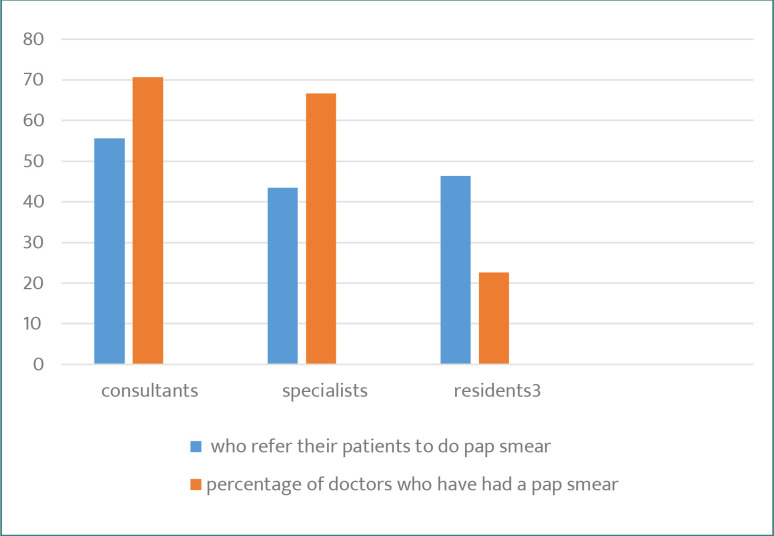
Practice of Pap smear according to job position

A higher proportion of consultants (66%) had undergone cervical cancer screening compared to specialists (33%). In addition, 55% of consultants and 54% of specialists referred their patients for cervical screening (Table 5C). Only 20% of residents had taken a Pap smear examination themselves, and 45% referred patients to do a Pap test examination. Figure 6 shows a comparison among the three groups of PHCCs female physicians toward practicing and promoting Pap smear tests.

## DISCUSSION

The results of our study demonstrate that a significant proportion of female doctors in PHCCs have adequate knowledge of cervical cancer screening. Specifically, almost 77% of physicians accurately answered questions related to Pap smear tests and related details. When we compared the knowledge levels among different groups of gynecologists, we found that specialists had the highest level of knowledge (87%), while residents had the lowest (70.74%). Interestingly, consultants, who are typically higher up in the hierarchy of any specialty, had lower knowledge levels than specialists, possibly due to a lack of recent review of theoretical knowledge after graduation from the medicine program.

Our findings differ from those of a previous study conducted by Sait *et al*. in 2009, which reported that only 35% of gynecological and 65% of non-gynecological doctors working at KAUH had appropriate knowledge of Pap smear screening [17]. In contrast, our study found that 98% of female gynecologists in PHCCs had knowledge of PST. There was a significant difference (p= 0.02) in the level of knowledge among participants. More than 90% of specialists were aware of the recommended age for initiating cervical cancer screening, whereas only 66% of consultants and 51% of residents knew this information. In addition, we found a significant difference (p=0.04) in knowledge about HPV as a cause of cervical cancer, with only 73% of residents being aware of this compared to 92% of specialists and 100% of consultants.

Despite the positive attitudes of all participants towards cervical cancer screening in the community, only 49.5% referred their patients for a PST. This is higher than the 35% reported in a previous study [17]. Another Ethiopian study that assessed the awareness and practice of cervical cancer screening among health workers found that 17% of the staff had taken the screening test for cervical cancer [19]. Furthermore, a study conducted in Ethiopia established that only 11.4% of healthcare workers received the screening test, despite having a higher level of knowledge about cervical cancer [20].

When investigating the factors that act as barriers to carrying out cervical cancer screening, we found that 24% of the respondents in our study reported not having enough time to do the referral, compared to 60% of doctors in Sait's study [17]. Moreover, 49.5% of our participants did not refer women for cervical cancer screening because of the absence of gynecological symptoms. In a study conducted in a University Hospital in Jeddah, doctors insisted that there were no clear guidelines for referring patients for a Pap test [17].

Another KAP study on female healthcare (HC) workers in Chennai Corporation, India, showed that while participants had good knowledge (100%) regarding cervical cancer screening and risk factors, 42% believed they were not at risk of developing the disease. Moreover, the absence of symptoms and risk factors were the main reasons chosen by the healthcare providers. The reasons for this low screening rate varied, with some participants citing a lack of awareness or symptoms, while others reported not having enough time or feeling uncomfortable with the screening process. The authors of the study emphasized that while knowledge is certainly important in promoting cancer screening, it is not always enough to motivate people to take action [21].

Doctors reported lack of time (47%), being asymptomatic (46%), and believing they were not at risk (26%) as reasons for not getting screened. Alternative low-tech techniques like Visual Inspection with Acetic acid (VIA) and Visual Inspection with Lugol's Iodine (VILI) have been recommended for married women over 35 years old, as they can be used as independent primary screening tests with immediate results, cutting down on turnaround time for histopathological expertise and allowing for further investigation and treatment the same day [22].

33% of the respondents had undergone the screening procedure compared to 40.7% of women in the Kingdom of Bahrain who have been screened at least once in their lifetime [8]. Furthermore, a study in the UAE found that while participants had limited knowledge about Pap smear, 54% had been screened at least once, with 31% screened twice and 18% screened three times [23]. The factors that led to a low uptake of Pap smear included lack of awareness (29%) and absence of symptoms (27%) [24]. 54% of women in Japan who took a PST took an internet-based survey and reported that it was related to higher income and employment status [24].

A previous systematic review and meta-analysis conducted in 2013 [25] investigated the factors influencing healthcare providers' (HCPs) adherence to cervical cancer screening guidelines using Pap smear. Lack of knowledge (82%) alongside negligence and misbeliefs (82%), followed by psychological reasons (73%), lack of time or cost (36%), lack of infrastructure or training factors (45%), and not giving any reasons (36%) were identified as the major factors. In the current study, around one-third of respondents did not provide any reasons to explain their lack of compliance with international guidelines for cervical cancer screening.

Self-HPV sampling has been tested in studies as a new approved screening tool that provides privacy and convenience for women to perform the test themselves at a time of their convenience without embarrassment [26]. Self-obtained HPV-positive tests can also provide under-screened women the opportunity to visit the clinic for follow-up diagnosis and management. Randomized control trials conducted in Norway and the United Kingdom included women who did not appear for their screening appointment or respond to second reminders. Their results support the use of self-sampling at home, which led to increasing follow-up attendance [26,27]. We can improve the screening practices in our community by using the self-help HPV sampling kit, especially if we want to fill the void created by the absence of a cervical cancer screening program.

A study conducted in the UK in 2017 investigated the factors that influence the client's decision to respond to an invitation for a screening test. The study concluded that the relationship between health providers and clients is essential to enhance the screening program. Trust, discussion of risks and benefits, and understanding fears and emotions are key to improving the effectiveness of screening [28,29].

Our recommendations include the initiation of a community-based educational program, taking into account the religious and cultural values of the population. Opportunistic screening in all clinics, with a clear explanation of the test and the importance of follow-up, is essential. Additionally, all women who visit gynecology or obstetrician clinics should be screened. The higher authorities may consider a national screening program, including the use of HPV self-sampling methods. It is also important to establish a fast-track, private, and confidential employee clinic that provides women's health services, including Pap smears for female doctors. Furthermore, the effectiveness of low-technology approaches like VIA and VILI should be investigated as a quicker primary screening test, and the utilization of Pap smears and biopsies as confirmatory diagnostic tests should be assessed. Finally, there is a need to explore the correlation between male circumcision and cervical cancer in our population, as most of the studies on this topic were done in the West.

## CONCLUSION

This study aimed to investigate the knowledge, attitude, and practice of cervical cancer screening among healthcare providers and also identify the barriers that hinder their adherence to screening guidelines. Overall, our findings suggest that healthcare providers have good knowledge and positive attitudes toward cervical cancer screening. However, non-compliance to screening guidelines can be attributed to misconceptions about symptoms, risk factors, and time constraints. Considering the cultural and religious beliefs in the community, it is important to acknowledge and address misconceptions surrounding cervical cancer screening, such as the belief that it is related to sexual practices outside of marriage or could result in loss of fertility. These myths and taboos need to be further assessed and addressed in community-based educational programs.

The low uptake of cervical cancer screening remains a public health challenge worldwide. It is crucial to involve mothers, wives, and community leaders in increasing awareness and promoting cervical cancer screening, emphasizing its importance in maintaining overall health and well-being. Furthermore, the use of self-help HPV sampling kits could potentially improve screening practices and increase compliance. Addressing misconceptions, promoting awareness, and providing access to screening services are essential steps in reducing the burden of cervical cancer.

## References

[1] Arbyn M, Weiderpass E, Bruni L, de Sanjosé S (2020). Estimates of incidence and mortality of cervical cancer in 2018: a worldwide analysis.. The Lancet Global Health..

[2] Ortiz AP, Soto-Salgado M, Calo WA, Hull P (2021). Elimination of cervical cancer in U.S. Hispanic populations: Puerto Rico as a case study.. Preventive Medicine..

[3] Bouvard V, Wentzensen N, Mackie A, Berkhof J (2021). The IARC Perspective on Cervical Cancer Screening.. The New England Journal of Medicine.

[4] Sung H, Ferlay J, Siegel RL, Laversanne M, Soerjomataram I, Jemal A, Bray F (2021). Global Cancer Statistics 2020: GLOBOCAN Estimates of Incidence and Mortality Worldwide for 36 Cancers in 185 Countries.. CA Cancer J Clin.

[5] Petignat P, Roy M (2007). Diagnosis and management of cervical cancer.. BMJ.

[6] Muñoz N, Bosch FX, de Sanjosé S, Herrero R (2003). Epidemiologic classification of human papillomavirus types associated with cervical cancer.. N Engl J Med.

[7] Alsbeih G, Ahmed R, Al-Harbi N, Venturina LA (2011). Prevalence and genotypes' distribution of human papillomavirus in invasive cervical cancer in Saudi Arabia.. Gynecologic Oncology.

[8] Li S, Wen X (2017). Seropositivity to herpes simplex virus type 2, but not type 1 is associated with cervical cancer: NHANES (1999–2014).. BMC Cancer.

[9] Fonseca-Moutinho JA (2011). Smoking and cervical cancer.. International Scholarly Research Network..

[10] Plummer M, Herrero R, Franceschi S, Meijer CJ (2003). Smoking and cervical cancer: pooled analysis of the IARC multi-centric case--control study.. Cancer Causes Control.

[11] Eze JN, Emeka-Irem EN, Edegbe FO (2013). A six-year study of the clinical presentation of cervical cancer and the management challenges encountered at a state teaching hospital in southeast Nigeria.. Clin Med Insights Oncol.

[12] Bruni L, Albero G, Serrano B, Mena M ICO/IARC Information Centre on HPV and Cancer (HPV Information Centre).. Human Papillomavirus and Related Diseases in the World. Summary Report 22 October 2021..

[13] Jassim G, Obeid A, Al Nasheet HA (2018). Knowledge, attitudes, and practices regarding cervical cancer and screening among women visiting primary health care Centres in Bahrain.. BMC Public Health.

[14] Almobarak AO, Elbadawi AA, Elmadhoun WM, Elhoweris MH, Ahmed MH (2016). Knowledge, Attitudes and Practices of Sudanese Women Regarding the Pap Smear Test and Cervical Cancer.. Asian Pac J Cancer Prev..

[15] Eze GU, Obiebi IP, Umuago IJ (2018). Perspectives of cervical cancer and screening practices among staff of a teaching hospital in South-South Nigeria.. Journal of Cancer Research and Practice.

[16] Al Khudairi H, Abu-Zaid A, Alomar O, Salem H (2017). Public awareness and knowledge of pap smear as a screening test for cervical cancer among Saudi population in Riyadh City.. Cureus..

[17] Sait KH (2011). Knowledge, attitudes, and practices regarding cervical cancer screening among physicians in the Western Region of Saudi Arabia.. Saudi Medical Journal.

[18] Farahat FM, Faqih NT, Alharbi RS, Mudarris RI (2021). Epidemiological characteristics of cervical cancer in a tertiary care hospital, western Saudi Arabia. A retrospective record-based analysis from 2002-2018.. Saudi Medical Journal..

[19] Kress CM, Sharling L, Owen-Smith AA, Dasalegn D (2015). Knowledge, attitudes, and practices regarding cervical cancer and screening among Ethiopian health care workers.. International Journal of Women's Health.

[20] Dulla D, Daka D, Wakgari N (2017). Knowledge about cervical cancer screening and its practice among female health care workers in southern Ethiopia: a cross-sectional study.. International Journal of Women's Health..

[21] Anantharaman VV, Sudharshini S, Chitra A (2012). A cross-sectional study on knowledge, attitude, and practice on cervical cancer and screening among female health care providers of Chennai corporation.. Journal of Academy of Medical Sciences..

[22] Badar F, Anwar N, Meerza F, Sultan F (2007). Cervical carcinoma in a Muslim community.. Asian Pacific Journal of Cancer Prevention.

[23] AL-Hammadi FA, Al-Tahri F, Al-Ali A, Nair SC, Abdulrahman M (2017). Limited understanding of pap smear testing among women, a barrier to cervical cancer screening in the United Arab Emirates.. Asian Pacific Journal of Cancer Prevention: APJCP.

[24] Kaneko N (2018). Factors associated with cervical cancer screening among young unmarried Japanese women: results from an internet-based survey.. BMC Women's Health.

[25] Asonganyi E, Vaghasia M, Rodrigues C, Phadtare A (2013). Factors affecting compliance with clinical practice guidelines for pap smear screening among healthcare providers in africa: systematic review and meta-summary of 2045 individuals.. PLoS One..

[26] Kitchener H, Gittins M, Cruickshank M, Moseley C (2018). A cluster randomized trial of strategies to increase uptake amongst young women invited for their first cervical screen: The STRATEGIC trial.. J Med Screen.

[27] Enerly E, Bonde J, Schee K, Pedersen H (2016). Self-sampling for human papillomavirus testing among non-attenders increases attendance to the Norwegian cervical cancer screening programme.. PloS One.

[28] Young B, Bedforf L, Kendrick D, Vedhara K (2018). Factors influencing the decision to attend screening for cancer in the UK: a meta-ethnography of qualitative research.. Journal of Public Health.

[29] Syler LB, Stobaugh CL, Foulis PR, Carlton GT (2021). Cervical Cancer Screening in South Florida Veteran Population, 2014 to 2020: Cytology and High-Risk Human Papillomavirus Correlation and Epidemiology.. Cureus..

